# Determinants of good academic performance among university students in Ethiopia: a cross-sectional study

**DOI:** 10.1186/s12909-022-03461-0

**Published:** 2022-05-23

**Authors:** Mesfin Tadese, Alex Yeshaneh, Getaneh Baye Mulu

**Affiliations:** 1 Department of Midwifery, College of Medicine and Health Sciences, Debre Berhan University, Debre Berhan, Ethiopia; 2 Department of Midwifery, College of Medicine and Health Sciences, Wolkite University, Wolkite, Ethiopia; 3 Department of Nursing, College of Health Sciences, Debre Berhan University, Debre Berhan, Ethiopia

**Keywords:** Academic performance, Predictors, Smoking, Ethiopia

## Abstract

**Background:**

Education plays a pivotal role in producing qualified human power that accelerates economic development and solves the real problems of a community. Students are also expected to spend much of their time on their education and need to graduate with good academic results. However, the trend of graduating students is not proportional to the trend of enrolled students and an increasing number of students commit readmission, suggesting that they did not perform well in their academics. Thus, the study aimed to identify the determinants of academic performance among university students in Southern Ethiopia.

**Method:**

Institution-based cross-sectional study was conducted from December 1 to 28, 2020. A total of 659 students were enrolled and data was collected using a self-administered questionnaire. A multistage sampling technique was applied to select study participants. Data were cleaned and entered into Epi-Data version 4.6 and exported to SPSS version 25 software for analysis. Bivariable and multivariable data analysis were computed and a *p*-value of ≤0.05 was considered statistically significant. Smoking, age, and field of study were significantly associated with academic performance.

**Result:**

Four hundred six (66%) of students had a good academic performance. Students aged between 20 and 24 years (AOR = 0.43, 95% CI = 0.22-0.91), and medical/ health faculty (AOR = 2.46, 95% CI = 1.45-4.20) were significant associates of good academic performance. Students who didn’t smoke cigarettes were three times more likely to score good academic grades compared to those who smoke (AOR = 3.15, 95% CI = 1.21-7.30).

**Conclusion:**

In this study, increased odds of good academic performance were observed among students reported to be non-smokers, adults, and medical/health science students. Reduction or discontinuation of smoking is of high importance for good academic achievement among these target groups. The academic environment in the class may be improved if older students are invited to share their views and particularly their ways of reasoning.

## Background

Higher education institutions play a pivotal role in producing qualified human power that enables solving the real problems of a community [1]. Education is a powerful agent of change that improves health and livelihoods and contributes to social stability. At the micro-level, it is associated with better living standards for individuals through improved productivity; given that those who have received a higher education tend to have more economic and social opportunities. At the macro level, education builds well-informed and skilled human capital, which has been considered an engine of economic growth, that positively contributes to economic development [2]. However, gaining knowledge, attitudes, values, and skills through education is not a simple task; rather it is a long and challenging trip in life. Students are expected to spend much of their time studying and need to graduate with good academic results.

Academic performance/ achievement is the extent to which a student, teacher, or institution has attained their short or long-term educational goals and is measured either by continuous assessment or cumulative grade point average (CGPA) [3]. A correlational study among vocational high school students in Indonesia found that students who had good academic achievements have higher income, better employment benefits, and more advancement opportunities [4]. Besides, academically successful students have higher self-esteem and self-confidence, low levels of anxiety and depression, are socially inclined, and are less likely to engage in substance abuse, i.e., alcohol and khat [5]. However, a cross-sectional study in Malaysia in higher learning institutions reported that an increasing number of students still do not graduate on time, suggesting that they did not perform well in their studies [6].

Despite excessive government investment in education, most students fail to achieve good academic performance at all levels of education. A correlational study in Arba Minch University, South Ethiopia, reported that the trend of graduating students is not proportional to the trend of enrolled students and more students commit readmission due to poor academic performance [7]. This resulted in unemployment, poverty, drugs elicit, promiscuity, homelessness, illegal activities, social isolation, insufficient health insurance, and dependence. Additionally, a systematic review in India concluded that poor academic achievement causes significant stress to the parents and low self-esteem to the students [8]. It is also significantly associated with high anxiety scores among university students in Pakistan [3]. Further, in public schools in Pakistan, academic failure affects self-concept and leads to a feeling of disturbance and shock. In this way, students finally drop out of the education system at all [9].

Beyond the quality of schools, various personal and family factors, including socioeconomic factors, English ability, class attendance, employment, high school grades, and academic self-efficacy have been proposed to influence academic performance. Besides, other factors, i.e., teaching skills, study hours, family size, and parental involvement have an association with academic performance as well [2, 10]. A cohort study among university students in Australia concluded that aging does not impede academic achievement [11]. A secondary data analysis among fifth-grade students in Colorado showed that eating breakfast, normal body mass index, adequate sleep, and ≥ 5 days’ physical activity per week was significantly associated with higher cumulative grades [12]. A significant association was also found between joining the medical profession and good academic performance in Pakistan [13]. At Arba Minch University, students with a good academic record before campus entry were more likely to have academic success in higher education programs [7]. A descriptive study on Bahir Dar university students showed that the education status of parents and attending night club affect academic performance [14]. Also, a survey in Nigerian high schools indicated students whose parents were government employees achieved better performance [15]. However, the impact of these factors varies from region to region and differs in cities and rural areas. This might be due to diverse data measurement methods and quality or the context of each study.

One of the critical barriers to academic success is substance use. A cross-sectional study in the US among high school seniors showed that substance users had greater odds of skipping school and having low grades [16]. Similarly, a descriptive survey among primary school students in Jordan indicated that smoking affects children’s physical and mental development and reduces academic achievement. Smoking was considered a barrier to optimal learning [17]. A cross-sectional study among university students in Wolaita Sodo found that substance use (smoking, khat chewing, drinking alcohol, and having an intimate friend who uses substances) was significantly and negatively associated with students’ academic performance [18]. In Jordan Primary school students, smoking was more likely to impair cognitive development, and decrease attentiveness and memory. This in turn leads to difficulty in remembering information and verbal learning impairment [17].

Most of the previous studies focus on primary and secondary education levels and the problem is not well addressed at the university level. The poor performance of university students requests attention. Moreover, in Ethiopia, limited studies were done on this topic and it was complicated by confounding factors. Thus, this study intended to identify the predictors of academic performance among university students in Southern Ethiopia.

## Methods and materials

### Study design, setting, and period

This is an institution-based cross-sectional study conducted among Hawassa University students from December 1 – 28, 2020. The University is one of the oldest public and residential national universities found in Hawassa city, Sidama Region. It is located 278 km south of Addis Ababa on the Trans-African Highway for Cairo-Cape Town. By the year 2020/21, the university has enrolled 21,579 students: 7955 Females and 13,624 Males. In general, there is one Institute of Technology and 10 colleges that offer 81 undergraduate, 108 Master’s, and 16 Ph.D. programs.

### Sample size and eligibility criteria

The sample size was calculated using Open Epi version 3.03 statistical software using percent of controls exposed (58%), odds ratio 0.63 [19], 80% power, and 95% confidence interval. By considering a 5 % non-response rate the final sample size was 659. All students who undergo their education in the selected departments and are available at the time of data collection were included in the study. Non-regular students, mentally and physically incompetent, and those who were not willing to fill out the questionnaire were excluded.

### Sampling procedure

The study was conducted among Regular Hawassa University students. A multi-stage sampling technique was applied to select study participants. The simple random sampling (SRS) technique was used to select representative colleges and departments. Students were stratified based on their batch/academic year. The sample size was distributed using probability proportional to size (PPS). Thereafter, SRS was applied to pick the required sample size from the predetermined sampling frame.

### Variables

Academic performance was the dependent variable. Independent variables include sociodemographic characteristics (age, gender, residence, parents’ education, family size, and faculty), individual factors (study hours, working after school, English language proficiency, sleeping hour, missing class, and entrance exam score), lifestyle and behavioral factors (substance use, breakfast, attending night club, and physical activity), and family and psychosocial variables (parents’ occupation, weight loss, and parent’s involvement).

### Data collection tool and quality control

The data was collected using a structured, self-administered questionnaire. Four data collectors and two supervisors participated in data collection. The questionnaire was prepared by reviewing similar published articles [2, 7, 20]. It was translated from English to the local language, Amharic, and then back to English by an independent translator to keep the consistency of the tool. Pre-testing was done on 5 % of the samples (33 students) at Dilla University and necessary adjustments were considered following the result (i.e., ethnicity, income). The principal investigators trained data collectors and supervisors about the objective and procedure of the study. The data were daily checked for completeness, consistency, and clarity.

### Measurement

#### Academic performance

Students who scored a cumulative GPA of 2.75 and above were categorized as “Good”, whereas those with a cumulative GPA of below 2.75 were categorized as “Poor” [7].

#### Habits

Participants who smoke at least one cigarette per day will be evaluated as smokers, and those who use more than one drink per day (any type of alcohol) will be considered alcohol consumers. Similarly, those who consume at least four glasses of tea and three cups of coffee per day will be accepted as those consuming tea and coffee, respectively [21].

#### Sugar intake

Excessive if individuals took 12 or more teaspoons of table sugar daily, moderate if 6 to 12 teaspoons; and restricted use if less than 6 teaspoons [22].

#### Extracurricular activities

Participation in school-based activities, i.e., sports, arts, and academic clubs [23].

### Data management and analysis

Data were cleaned and entered into Epi-Data version 4.6 and SPSS statistical package version 25 was applied to perform all the statistical analysis. Cross-tabulation of variables was computed and the Chi-Square (X^2^) test was used to analyze the variables. Pearson Chi-Square test was reported for variables that fulfill the assumption of the X^2^ test. Whereas Fisher’s Exact Test was reported for variables having an expected count of less than five. Bivariable and multivariable logistic regression analysis were performed to identify independent predictors of academic performance. Variables with a *p*-value of ≤0.25 in the bivariable logistic regression were included in the final model. Descriptive statistics were used to describe the characteristics of participants. Adjusted odds ratios (AORs) with 95% confidence intervals were used to interpret the strength of association, and the Hosmer-Lemeshow goodness-of-fit was used to check for model fitness. A two-tailed *p*-value of ≤0.05 was considered to declare statistically significant.

## Result

### Baseline characteristics of participants

Six hundred fifteen (615) students were involved in the study, making a 93.3% response rate. The age of students ranged from 18 to 29 years with the mean age of 21.62 ± 1.89 and 21.73 ± 2.08 for academically poor and good students, respectively. About 39% of rural residents had poor academic performance (PAP), whereas 69.3% of urban residents had good academic performance (GAP) (*p* = 0.035). Further, more than one-third (38.9%) of non-medical/non-health students and 82.9% of medical/health students scored PAP and GAP, respectively (*p* < 0.00) (Table 1).

**Table 1:** Baseline characteristics of participants, Southern Ethiopia

Variables	Category	Academic Performance		***p***-value
		Poor	Good	
**Age**	15 - 19 years	9 (29.0)	22 (71.0)	0.066^1^
	20 – 24 years	187 (35.8)	336 (64.2)	
	25 – 29 years	13 (21.3)	48 (78.7)	
**Gender**	Male	109 (29.7)	258 (70.3)	0.579^1^
	Female	93 (37.5)	155 (62.5)	
**Residence**	Rural	94 (39.0)	147 (61.0)	0.035^1*^
	Urban	115 (30.7)	259 (69.3)	
**Mothers’ education**	No formal education	113 (39.2)	175 (60.8)	0.005^1*^
	Primary	36 (34.6)	68 (65.4)	
	Secondary	33 (34.4)	63 (65.6)	
	College and above	27 (21.3)	100 (78.7)	
**Fathers’ education**	No formal education	92 (40.5)	135 (59.5)	0.051^1*^
	Primary	32 (31.4)	70 (68.6)	
	Secondary	27 (33.8)	53 (66.3)	
	College and above	58 (28.2)	148 (71.8)	
**Faculty**	Medical/Health	24 (17.1)	116 (82.9)	< 0.00^1*^
	Non-medical/Health	185 (38.9)	290 (61.1)	
**Family size**	Small	30 (28.3)	76 (71.7)	0.304^1^
	Medium	169 (35.6)	306 (64.4)	
	Large	10 (29.4)	24 (70.6)	

^1^Two-sided Pearson Chi-Square Test

^1*^p-value ≤0.25

### Family and psychosocial characteristics

As shown in Table two below, 34% of students who experience weight loss scored poor academic results, while 66% of students who didn’t experience weight loss scored good academic results. Additionally, 38.7% of students who belong to agriculturalist families registered poor academic points, whereas 69.7% of students who belong to government employees scored academically good results (Table 2).

**Table 2:** Family and psychosocial characteristics of participants, Southern Ethiopia

Variables	Category	Academic performance		***p***-value
		Poor	Good	
**Weight loss within the past one-year**	Yes	39 (33.9)	76 (66.1)	0.986^1^
	No	170 (34.0)	330 (66.0)	
**Mothers’ occupation**	Housewife	138 (35.9)	246 (64.1)	0.082^1*^
	Gov’t employee	24 (24.2)	75 (75.8)	
	Self-employed	47 (35.6)	85 (64.4)	
**Fathers’ occupation**	Farmer	89 (38.7)	141 (61.3)	0.152^1*^
	Gov’t employee	56 (30.3)	129 (69.7)	
	Self-employed	64 (32.0)	136 (68.0)	
**Parent’s involvement**	Yes	199 (34.4)	379 (65.6)	0.357^1^
	No	10 (27.0)	27 (73.0)	

^1^Two-sided Pearson Chi-Square Test

^1*^p-value ≤0.25

### Behavioral characteristics

One-third (67%) of students involved in regular physical activity scored GAP. About 58.8% of students who smoke cigarettes had PAP, whereas 66.7% of students who didn’t smoke scored GAP (chi^2^ *p* = 0.028). Additionally, 35% of students who attend night club scored PAP, while 66.2% of students who didn’t attend night club scored GAP (Table 3).

**Table 3:** Behavioral characteristics of participants, Southern Ethiopia

Variables	Category	Academic performance		***p***-value
		Poor	Good	
**Physical activity**	Yes	47 (33.1)	95 (66.9)	0.800^1^
	No	162 (34.2)	311 (65.8)	
**Alcohol consumption**	Yes	69 (37.7)	114 (62.3)	0.205^1*^
	No	140 (32.4)	292 (67.6)	
**Smoking**	Yes	10 (58.8)	7 (41.2)	0.028^1*^
	No	199 (33.3)	399 (66.7)	
**Khat use**	Yes	11 (55.0)	9 (45.0)	0.044^1*^
	No	198 (33.3)	397 (66.7)	
**Breakfast per week**	Not at all	3 (15.0)	17 (85.0)	0.186^2^
	< 5 days	56 (33.9)	109 (66.1)	
	≥ 5 days	150 (34.9)	280 (65.1)	
**Teaspoons of sugar used per day**	No at all	39 (33.9)	76 (66.1)	0.682^2^
	Minimal	143 (33.3)	287 (66.7)	
	Moderate	25 (40.3)	37 (59.7)	
	Excessive	2 (25.0)	6 (75.0)	
**Attend night club/party**	Yes	29 (34.9)	54 (65.1)	0.843^1^
	No	180 (33.8)	352 (66.2)	

^1^Two-sided Pearson Chi-Square Test

^1*^p-value ≤0.25

^2^Two-sided Fisher’s Exact Test

### Personal characteristics

A higher proportion of participants who studied more than 4 hours per day (69.3%) scored GAP. One-third (35.4%) of students who sleep more than 7 hours per night registered PAP, while 68.4% of students who sleep less than 7 hours scored GAP. About 46.2% of students who had a pre-intermediate level of English proficiency were poor in academics, whereas 80.6% of students with an advanced level of proficiency were good in academics (chi^2^ *p* = 0.002) (Table 4).

**Table 4:** Personal characteristics of students, Southern Ethiopia

Variables	Category	Academic performance		***p***-value
		Poor	Good	
**Study hours per day**	≤ 4 hours	98 (38.7)	155 (61.3)	0.038^1*^
	> 4 hours	111 (30.7)	251 (69.3)	
**Work after school or employed**	Yes	16 (26.7)	44 (73.3)	0.208^1*^
	No	193 (34.8)	362 (65.2)	
**Sleeping hours**	Inadequate (< 7 hrs.)	75 (31.6)	162 (68.4)	0.332^1^
	Adequate (≥7 hrs.)	134 (35.4)	244 (64.6)	
**English proficiency**	Elementary level	39 (38.2)	63 (61.8)	0.002^1*^
	Pre-intermediate level	49 (46.2)	57 (53.8)	
	Intermediate level	100 (32.9)	204 (67.1)	
	Upper-intermediate level	15 (20.8)	57 (79.2)	
	Advanced level	6 (19.4)	25 (80.6)	
**Class absentees per semester**	No absentee	117 (30.8)	263 (69.2)	0.038^2^
	≤15 days	87 (38.3)	140 (61.7)	
	≥16 days	5 (62.5)	3 (37.5)	
**Entrance exam score**	Very good	1 (6.7)	14 (93.3)	< 0.00^2^
	Good	31 (16.8)	154 (83.2)	
	Satisfactory	177 (42.7)	238 (57.3)	

^1^Two-sided Pearson Chi-Square Test

^1*^p-value ≤0

^2^Two-sided Fisher’s Exact Test

### Academic performance

Overall, 406 (66%) of students had a good academic performance. The mean CGPA of students was 2.92 (SD ± 0.48), with a minimum of 1.80 and a maximum of 4.00 points. The mean CGPA of academically poor students was 2.39 points, which is lower by 0.81 compared to academically good students (3.20 points).

### Determinants of academic performance

In the multivariable logistic regression analysis, age, faculty, and smoking have shown a statistically significant association with academic performance (Table 5).

**Table 5:** Determinants of academic performance, Southern Ethiopia

Variables	Category	COR (95% CI)	AOR (95% CI)	***p***-value
**Age**	15 - 19 years	0.66 (0.25-1.78)	0.49 (0.16-1.45)	0.196
	20 – 24 years	0.49 (0.26-0.92)	0.43 (0.22-0.91)	0.026^*^
	25 – 29 years	1	1	
**Residence**	Rural	1	1	0.838
	Urban	1.44 (1.03-2.02)	0.95 (0.58-1.54)	
**Mothers’ education**	No formal education	1	1	
	Primary	1.22 (0.76-1.95)	0.97 (0.54-1.74)	0.912
	Secondary	1.23 (0.76-1.99)	1.01 (0.49-2.12)	0.963
	College and above	2.39 (1.47-3.89)	1.58 (0.66-3.77)	0.299
**Fathers’ education**	No formal education	1	1	
	Primary	1.49 (0.91-2.45)	1.41 (0.81-2.45)	0.227
	Secondary	1.34 (0.78-2.28)	1.05 (0.51-2.16)	0.900
	College and above	1.74 (1.16-2.60)	1.28 (0.60-2.71)	0.522
**Faculty**	Medical/Health	3.08 (1.91-4.96)	2.46 (1.45-4.20)	0.001^*^
	Non-medical/Health	1	1	
**Mothers’ occupation**	Housewife	1	1	
	Gov’t employee	1.75 (1.06-2.90)	1.08 (−.53-2.21)	0.825
	Self-employed	1.01 (0.67-1.53)	0.76 (0.46-1.28)	0.310
**Fathers’ occupation**	Farmer	1	1	
	Gov’t employee	1.45 (0.96-2.19)	0.85 (0.44-1.64)	0.630
	Self-employed	1.34 (0.90-1.99)	1.09 (0.63-1.91)	0.755
**Alcohol consumption**	Yes	1	1	
	No	1.26 (0.88-1.81)	1.20 (0.81-1.79)	0.370
**Smoking**	Yes	1	1	
	No	3.86 (1.47-7.64)	3.15 (1.21-7.30)	0.025^*^
**Khat use**	Yes	1	1	
	No	2.45 (0.99-6.01)	1.91 (0.57-6.36)	0.292
**Breakfast**	Yes	0.33 (0.09-1.15)	0.40 (0.11-1.52)	0.179
	No	1	1	
**Study hours per day**	≤ 4 hours	1	1	
	> 4 hours	1.43 (1.02-2.00)	1.29 (0.90-1.85)	0.162
**Work after school or employed**	Yes	1	1	
	No	0.68 (0.37-1.24)	0.67 (0.35-1.28)	0.234
**English proficiency**	Elementary level	1	1	
	Pre-intermediate level	0.72 (0.41-1.25)	0.69 (0.39-1.26)	0.231
	Intermediate level	1.26 (0.79-2.01)	1.06 (0.64-1.75)	0.809
	Upper-intermediate level	2.35 (1.17-4.71)	1.80 (0.84-3.85)	0.130
	Advanced level	2.58 (0.97-6.85)	1.39 (0.47-4.11)	0.551

^*^statistically significant at *p*-value < 0.05

Students aged between 20 and 24 years were 56% less likely to score good academic performance compared to those who were aged between 25 and 29 years (AOR = 0.43, 95% CI = 0.22-0.91). Medical/ health science students were two times more likely to attain good academic points compared to their counterparts (AOR = 2.46, 95% CI = 1.45-4.20). Students who didn’t smoke cigarettes were three times more likely to register good academic grades compared to those who smoke (AOR = 3.15, 95% CI = 1.21-7.30).

## Discussion

This study investigated the determinants of academic performance. The finding showed that only two-thirds (66%) of university students score good academic grades. Age, faculty, and cigarette smoking were found to have a statistically significant association with academic performance.

Students who didn’t smoke cigarettes were more likely to register good academic grades compared to those who smoke. This is consistent with the findings observed among university students in western societies. Smoking cigarettes were associated with decreased odds of high academic achievement in Norwegian students [19]. A cohort study in England showed that tobacco use was strongly linked with subsequent adverse educational outcomes [24]. Similarly, in Jordan, lower academic performance was positively associated with smoking [17]. In both Pakistan [25] and Korea [26], students who achieve good academic performance were less likely to smoke. Besides, a study from Finland suggested that smoking both predicts and is predicted by lower academic achievement [27]. The use of substances including smoking is known for its significant association with mental distress and depression. It also increases the risk of respiratory infections, asthma, tuberculosis, certain eye diseases, and problems of the immune system as well as increases the risk of bacterial meningitis, especially among freshman living in dorms. Additionally, smoking had a great influence on the attitude, emotion, and behavior of students, and can motivate them to perform their bests. For instance, in Australia, 69% of smokers attended bars, nightclubs, or gaming venues at least monthly [28]*.* Further, smokers are substantially engaged in khat chewing and alcohol drinking [29]. Having a serious health complication, wasting study hours, and concomitant substance use in college might prevent students from being able to perform their best in school. This finding call attention on prevention efforts aimed at students to reduce the detrimental consequences on academic performance.

In this study, students aged between 20 and 24 years were less likely to score good academic performance compared to those who were aged between 25 and 29 years. This effect favors the older students. A comparable result was obtained in Australia. The study showed that aging does not impede academic achievement and discrete cognitive skills as well as lifetime engagement in cognitively stimulating activities promote academic success in adults [11]. Similarly, age was positively related to the CGPA of the students in Nigeria [30]. According to a cross-sectional study in Norway, higher age was associated with better average academic performance of students [31]. Older students, that is 25 years and above are wiser and more mature. Students of a higher age may have a stronger motivation for studying and follow a more productive approach to studying; that means, they may employ more deep and strategic approaches than surface approaches. Additionally, older have more life experience than younger ones. Older students may personally or by their relationships to others, have experience with failure and success, illness and recovery, and loneliness and companionship in a range of settings and domains. Experience with the bright and the dark sides of life, and reflecting on and learning from that experience may encourage students’ ability to apply a variety of theoretical perspectives for academic assignments. As a result, older students may benefit and achieve good academic results.

The current study found that medical/ health science students were more likely to attain good academic points compared to their counterparts. Similarly, in Pakistan, joining the medical profession was significantly linked with good academic scores [13]. Admission to medical school was also a significant predictor of good academic performance in Nigeria [32]. Additionally, in Southern Ethiopia, poor academic performance was significantly higher among agriculture students than health science students [33]. The possible explanation might be that medicals students have higher levels of stress than non-medical and this was mostly attributed to their studies (75.6%) [34]. The stress showed beneficial effects on medical students. Exam, test, and assignment-related stress was associated with high attendance, better day-to-day activities, and good academic results [35]. In addition, medical students had significantly higher intrinsic motivation for academics [36].

### Limitation

The study has some limitations. First, there might be social desirability bias as a result of self-administered data collection techniques. However, anonymity and confidentiality were assured. Second, some potential confounders, i.e., institutional influences were not controlled. Third, self-reporting may have resulted in under or over-reporting of some factors. Fourth, the cross-sectional nature does not allow the making of direct causal inferences.

### Implication

Education is a powerful agent of change that produces qualified human power, improves health and livelihoods, accelerates economic development, and solves the real problems of a community. Students are expected to spend much of their time on their education and need to graduate with good academic results. Academically good students have better employment benefits, higher income, higher self-esteem and self-confidence, low levels of anxiety and depression, and are less likely to engage in substance abuse. However, in this study, only two-thirds of university students achieved good academic grades. Smoking, age, and field of study were significantly associate with academic performance. The finding of the study had the academic implication that cessation of smoking had a paramount benefit for academic success, and hence more employment opportunities and good quality of life.

## Conclusion

Increased odds of good academic performance were observed among students reported to be non-smokers, adults, and medical/health science students. Reduction or discontinuation of smoking is of high importance for good academic achievement among these target groups. The finding suggests that higher university officials need to raise awareness regarding the adverse educational outcomes of smoking through public service announcements and curriculum-based education. Additionally, policies concerning smoking restrictions in community spaces and university facilities may help reduce the onset of smoking. The current action taken to promote a smoke-free student population can impact the future health of Ethiopians, future leaders, scholars, and professionals. Further, the academic environment in the class may be improved if older students are invited to share their views and particularly their way of reasoning. Although this study had provided some primary evidence, more similar studies documenting the association between tobacco use and academic performance among Ethiopian University students are warranted.

## Data Availability

The datasets used in the current study are available from the corresponding author and can be presented upon a reasonable request.

## Abbreviations

CGPA: Cumulative Grade Point Average
GAP: Good Academic Performance
PAP: Poor Academic Performance
SPSS: Statistical Package for Social Science
SRS: Simple Random Sampling
WHO: World Health Organization
